# Failure of androgenesis in *Miscanthus* × *giganteus* in vitro culture of cytologically unbalanced microspores

**DOI:** 10.1007/s00497-013-0219-6

**Published:** 2013-07-04

**Authors:** Iwona Żur, Ewa Dubas, Aneta Słomka, Franciszek Dubert, Elżbieta Kuta, Agnieszka Płażek

**Affiliations:** 1 Franciszek Górski Institute of Plant Physiology, Polish Academy of Sciences, Niezapominajek 21, 30-239 Krakow, Poland; 2 Department of Plant Physiology, University of Agriculture, Podłużna 3, 30-239 Krakow, Poland; 3 Department of Plant Cytology and Embryology, Jagiellonian University, Grodzka 52, 31-044 Krakow, Poland

**Keywords:** Anther culture, Honey, Meiotic abnormalities, Microspore embryogenesis

## Abstract

*Miscanthus* × *giganteus* is a popular energy crop, which due to its hybrid origin is only vegetatively reproduced. Asexual embryogenesis in anther and microspore culture leading to double haploids production could allow to regain the ability for sexual reproduction and to increase the biodiversity of the species. Therefore, the goal of this paper was to investigate the requirements of androgenesis in *Miscanthus*. The standard protocols used for monocotyledonous plants were applied with many modifications regarding the developmental stage of the explants at the time of culture initiation, stress treatment applied to panicles and isolated anthers as well as various chemical and physical parameters of in vitro culture conditions. Our results indicated that the induction of androgenesis in *M.* × *giganteus* is possible. However, the very low efficiency of the process and the lack of regeneration ability of the androgenic structures presently prevent the use of this technique.

## Introduction

*Miscanthus* × *giganteus* is a perennial grass of the *Poaceae* family that recently, due to its high biomass productivity and low nutritional requirements, has become a promising candidate for commercial biofuel/bioenergy production (Heaton et al. 2004, 2008). Cytological and molecular studies have revealed that it is a natural allotriploid (2*n* = 3x = 57) derived from a cross between diploid *M. sinensis* (2*n* = 2x = 38) and tetraploid *M. sacchariflorus* (2*n* = 4x = 76) (Greef and Deuter 1993; Linde-Laursen 1993; Hodkinson et al. 2002a, b, c; Swaminathan et al. 2010). This fact has been confirmed by the natural occurrence of *Miscanthus* triploid plants in southern regions of Japan (Nishiwaki et al. 2011). As a triploid, it is sterile (Den Nijs and Peloquin 1977); however, according to the ‘triploid bridge’ hypothesis (Jackson 1976; Wang et al. 2010 and references therein), the production of some fertile gametes is possible. In our earlier study (Słomka et al. 2012), the frequency of stainable pollen grains ranged from 13.9 to 55.3 % depending on the pollen staining method, but pollen germination was not observed either in vitro or *in planta*. The wide range of pollen diameters (25.5–47.6 μm) observed suggests irregular meiotic divisions. Based on the information from Den Nijs and Peloquin (1977) and Mendiburu and Peloquin (1976) that cell volume increases with increasing DNA content, it could be supposed that not all produced microspores were haploid.

From an agro ecological point of view, sterility can be an advantage, allowing for introduction of species into new environments without cross-pollination with native species (Hodkinson et al. 2002b). On the other hand, asexual propagation causes very low genetic diversity, as almost all European populations of *M.* × *giganteus* originated from the sample taken in 1935 by A. Olson in Yokohama, Japan (Greef et al. 1997). Such homogeneity makes genetic improvement impossible. Moreover, the high cost of vegetative or micropropagation significantly limits the cultivation of this highly valuable crop (Lewandowski 1998).

In this study, several questions were addressed: (1) Is it possible to induce normal haploid microspore development to produce doubled haploids (DHs) despite the disturbed meiosis? (2) Are cytologically unbalanced microspores capable of dividing and forming androgenic embryos in order to generate new genetic variations for breeding purposes? (3) Does the androgenic pathway resemble the zygotic embryogenesis?

The requirements for embryogenesis initiation in anther and microspore cultures of *M.* × *giganteus* were investigated. The standard protocols used for monocotyledonous plants were applied. Modifications were made to the developmental stage of the explants at the time of culture initiation, stress treatment applied to panicles and isolated anthers and various chemical and physical parameters of in vitro culture.

## Materials and methods

### Plant material

*Miscanthus* × *giganteus* rhizomes were obtained from the Institute of Plant Breeding and Acclimatization in Radzików near Warsaw (Poland). Some maternal plants were cultivated in a glasshouse in 15 l pots filled with commercial soil (pH = 5.8) at 25 °C and 65 % humidity under natural light, supplemented with light at 400 μmol m^−2^ s^−1^ from AgroPhilips lamps for a 12/12 h (day/night) photoperiod. Other plant material was originated from the Horticultural Farm in Zabierzów (located near to Kraków) and was grown in the experimental field belonging to the University of Agriculture (Kraków, Poland).

### Inflorescence pretreatment

The inflorescences were harvested at different developmental stages characterized by two morphological parameters: (a) the length (cm) between the base of the flag leaf and the penultimate leaf collar regions, and (b) the length (cm) of the panicle tip emerged from the sheath. Three anthers from the upper, middle and lower parts of a panicle were collected, and the viability and developmental stage of microspores were assessed (see below).

The inflorescences were wrapped in foil bags, placed immediately in Hoagland’s salt solution and stored for 7, 10, 14 or 21 days in the darkness at 4, 10, 15 or 20 °C. Subsequently, the spikes were sprayed with 70 % ethanol, surface sterilized in 20 % commercial bleach (‘Domestos’) solution for 15 min and then rinsed 4–5 times with sterile deionized water.

### Anther culture

Aseptically excised anthers were placed in 60 × 15 mm Petri dishes containing the following induction media: C17 (Wang and Chen 1986), KFWC (Kuhlmann and Foroughi-Wehr 1989) modified according to Sidhu and Davies (2009) or 190-2 (Zhuang and Xu 1983). The standard media were supplemented with 1 mg l^−1^ dicamba, 1 mg l^−1^ picloram and 0.5 mg l^−1^ kinetin, 90 g l^−1^ maltose and 0.6 % agar; pH 5.8. The effect of other hormonal compositions was also tested: (1) 2 mg l^−1^ 2,4-D and 0.5 mg l^−1^ kinetin, (2) 1 mg l^−1^ dicamba, 0.5 mg l^−1^ picloram and 0.5 mg l^−1^ kinetin and (3) 2 mg l^−1^ IBA and 0.5 mg l^−1^ kinetin. Moreover, with the use of standard C17 medium, the effect of maltose (90 g l^−1^) substitution with the same concentration of commercial honey (OSP Pszczelarz Krakow) was also tested. In other variants, the C17 and KFWC standard media were supplemented with 10, 50 or 100 mg l^−1^ arabinogalactan proteins (AGPs) (Arabic Gum from acacia tree, G9752 Sigma-Aldrich).

In three replications of the experiment, anthers extracted from panicles were inoculated in a pretreatment medium containing 40 mM l^−1^ CaCl_2_ 2H_2_O, 6 g l^−1^ agarose and 0.7, 1 or 1.5 M mannitol according to the method described by Cistué et al. (2003). The cultures were incubated at 28 or 32 °C in the dark for 2–7 days and then transferred to various variants of the induction media.

In three other replications, the effect of 0.1 % *n*-butanol (281549, Sigma-Aldrich) treatment according to Soriano et al. (2008) was evaluated. Isolated anthers were inoculated in 0.3 M mannitol or liquid 190-2 medium supplemented with 0.1 % *n*-butanol (v/v) and kept at 28 °C, in the dark for 6 or 24 h. Controls without *n*-butanol were performed. After washing in 0.3 M mannitol, the anthers were transferred to variants of the induction media.

In all experiments, the anthers were co-cultured with immature ovaries isolated from the same inflorescence (120 anthers and 20 ovaries *per* dish).

A procedure to prevent anther desiccation was also tested in five replications by transferring anthers dampened with sterile distilled water, B medium (Kyo and Harada 1986) or modified KFWC. Modified KFWC was supplemented with 1 mg l^−1^ dicamba, 1 mg l^−1^ picloram, 0.5 mg l^−1^ kinetin and 10 mg l^−1^ AGPs to induction media.

All cultures were incubated in the dark at 28 ± 1 °C. However, in some cultures (at least five biological replication), various preculture conditions were also studied: 24 h at 30 °C, or 24 h at 4 °C or 5 days at 4 °C followed by culture at standard conditions.

### Isolated microspore culture

Various procedures of microspore isolation were tested: (a) direct method with the use of Waring blender in which 2–3 cm panicle segments were blended in 0.3 M mannitol, or (b) indirect method composed of two steps: (1) anther isolation and preculture in B medium (Kyo and Harada 1986) or 190-2 medium for 1–3 days at 5, 26 or 32 °C followed by (2) microspore isolation with the use of magnetic stirring bar (Touraev and Heberle-Bors 2003) or by delicate squashing and pulping with a ceramic rod (Zur et al. 2008). The direct method was used only in the case of field-grown plants in which the inflorescences were well developed with many primary branches and a large number of spikelets. Plants grown in glasshouse produced smaller panicles with reduced number of branches and a very hard rachis, which excluded the use of a Waring blender. The indirect method consisted of anther extraction with the use of forceps followed by microspore isolation. Results show that the use of a ceramic rod for anther homogenization was more efficient. The resulting slurry obtained was filtrated through a 40 μm nylon membrane and pelleted (100×*g*, 7 min). After removing the supernatant, the microspores were resuspended in 0.3 M mannitol and gently layered onto a 21, 23, 25 or 30 % maltose solution for density gradient centrifugation (80×*g*, 7 min).

Viable microspores settled at the interface between mannitol and maltose were collected, washed in 0.3 M mannitol and centrifuged again (100×*g*, 7 min). The supernatant was removed, and the pelleted microspores were resuspended again in 1 ml 0.3 M mannitol. The total number of collected microspores was estimated by microscopic observation using a Neubauer counting chamber. Induction medium was added to produce a final culture density of 100,000 microspores *per* ml.

For androgenesis induction, liquid 190-2 or KFWC media were used with some modifications: (1) 500 mg l^−1^ casein hydrolysate (CH), 60 g l^−1^ maltose, pH 5.8 (224 mOsmol kg^−1^); (2) 500 mg l^−1^ CH, 90 g l^−1^ maltose, pH 5.8 (317 mOsmol kg^−1^); (3) 500 mg l^−1^ CH, 60 g l^−1^ maltose, 0.375 mg l^−1^ 2,4-D, 0.125 mg l^−1^ kinetin, pH 5.8 (246 mOsmol kg^−1^); (4) 500 mg l^−1^ CH, 60 g l^−1^ honey (OSP Pszczelarz Krakow), pH 5.8 (341 mOsmol kg^−1^).

The microspore suspensions were plated in 35 × 15 mm Petri dishes (1.5 ml per dish) and co-cultured with immature ovaries (10 per 1.5 ml of cell’ suspension) that were dissected from the same cold-treated inflorescences. The cultures were incubated in darkness at 26 °C or in some (at least three) replicates precultured for 1, 2 or 3 days at 5 °C or 32 °C and then transferred to 26 °C.

### Regeneration

Androgenic structures (AS) of ≈1 mm size were transferred onto 0.6 % agar solidified modified regeneration medium 190-2 (Zhuang and Xu 1983) containing 0.5 mg l^−1^ kinetin, 0.5 mg l^−1^ NAA and 3 % sucrose. The cultures were kept at 26 °C in dim light [80–100 μmol m^−2^ s^−1^ (PAR)] with a 16/8 h (day/night) photoperiod.

### Cytological observations light microscopy

Microscopic analyses were performed on the day of isolation and then on the 3rd, 7th, 10th, 14th, 21th and 28th days of in vitro culture. Cell morphology was examined in Petri dishes containing suspension culture with an inverted light microscope (NIKON TS-100/100F) with Hoffman contrast. The percentages of cells and structures with different features were calculated over a total of 500 objects per analysis. The experiment was based on eight biological replicates (each Petri dish was considered one biological replicate).

Microscope slides were examined under a Nikon Eclipse-E600 equipped with a differential interference contrast (DIC) system. Images were collected with a Nikon DS-Ri1 digital camera and processed with NIS-Elements AR 3.0 Imaging Analysis, Microsoft Power Point and Corel PhotoPaint 10.0.

### Fluorescence microscopy

Chromatin was stained with 4′6-diamidino-2-phenylindole * 2HCl (DAPI) (0.0001 %) (λ_Ex_ = 365 nm; λ_Em_ = 420 nm, blue fluorescence) according to Custers et al. (1994). Samples of isolated anthers containing microspores were collected in Eppendorf tubes and incubated independently in DAPI, and then anthers were placed on slides, squashed and analyzed.

Microspore viability was determined by fluorochromatic reaction to fluorescein diacetate (FDA) (0.01 %) (λEx = 465 nm, λEm = 515 nm, green fluorescence) (Heslop-Harrison and Heslop-Harrison 1970) in samples on the isolation day. Samples of freshly isolated microspores/pollen suspension (100 μl) were collected in Eppendorf tubes and incubated in FDA, and then a drop of suspension was placed on a microscope slide and analyzed.

FDA was made up as stock solution in acetone at 5 mg ml^−1^. Immediately before use, dilutions were prepared by adding drops of the stock to 2 ml 15 % sucrose solution until saturation was reached, as indicated by the appearance of persistent turbidity.

### Scanning electron microscopy (SEM)

Dry pollen grains, isolated from air-dried inflorescences, were dusted onto stubs with caronto stubs with carbon-conductive double-sided adhesive disks (SPISupplies, Structure Probe, Inc., Chester, PA, USA), gold-coated and examined with a HITACHI S-4700 SEM in the Scanning Microscopy Laboratory of Biological and Geological Sciences of the Jagiellonian University.

## Results

### Optimal stage for microspore isolation to induce androgenesis

**Fig. 1:**
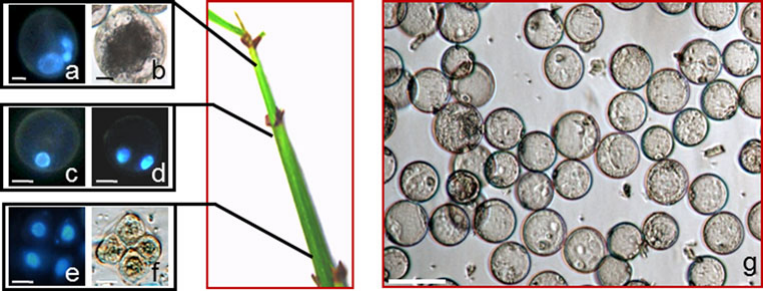
Morphological and cytological characteristic of *Miscanthus* × *giganteus* microspores. Tri-cellular pollen grain (**a**), pollen grain filled with starch grains (**b**), late uni-nucleate microspore (**c**), bi-cellular pollen grain (**d**), tetrads (**e, f**). **g** Viable, isolated microspores in suspension at day 0 of culture. Blue fluorescence (UV) of DAPI demonstrates nuclei (**a, c–e**). *Bar* 10 μm

Morphological observations combined with cytological analyses of pollen development indicated that the microspores at the optimal developmental phase for androgenesis induction were found in panicles enclosed by the flag leaf when the collar region of the flag leaf had come 4–7 cm out of the penultimate leaf, and the tip of the inflorescence had emerged up to 3 cm (Fig. 1). The development of microspores was not synchronized across the panicle; therefore, meiocytes at different stages of meiosis, microspore tetrad, microspores and 2–3 nucleate pollen grains could be found. In the lower part of a panicle, tetragonal tetrads of microspores were surrounded by callose deposits forming a thick visible wall (Fig. 1a–g). In the upper region of a panicle, uni-nucleated, non-vacuolated microspores were noted with the nucleus located in the center of dense cytoplasm. In the middle part of the inflorescence, light green anthers 2.3–2.5 mm long, with vacuolated microspores that had the nucleus located at one pole, the best stage for androgenesis induction, occurred with a highest frequency of 91.3 %.

Panicle pretreatments lasting more than 2 weeks at 4 °C increased the percentage of degenerated microspores (93 %). The highest percentage of viable microspore was noted when the panicles were pretreated for 7 days at 4 °C (55–66 %) or for 14 days at 10 °C (53–69 %).

### Androgenesis induction in anther cultures

Extensive anther tissue browning was observed in all cultures regardless of the type and duration of pretreatment or culture conditions. In a few cases, single androgenic structures (in <0.1 % of the anthers) were produced on: (1) C17 medium supplemented with 2 mg l^−1^ 2,4-D, 0.5 mg l^−1^ kinetin after 7 days at 4 °C followed by 24 h preculture at 32 °C and (2) C17 medium supplemented with 1 mg l^−1^ dicamba, 1 mg l^−1^ picloram and 0.5 mg l^−1^ kinetin with the use of an antidesiccation procedure (liquid KFWC containing 1 mg l^−1^ dicamba, 1 mg l^−1^ picloram, 0.5 mg l^−1^ kinetin, 10 mg l^−1^ AGPs) after pretreatment of 14 days at 10 °C.

The androgenic structures had no regeneration ability and degenerated after a few weeks of culture on modified regenerating medium 190-2.

### Androgenesis induction in isolated microspore cultures

**Fig. 2:**
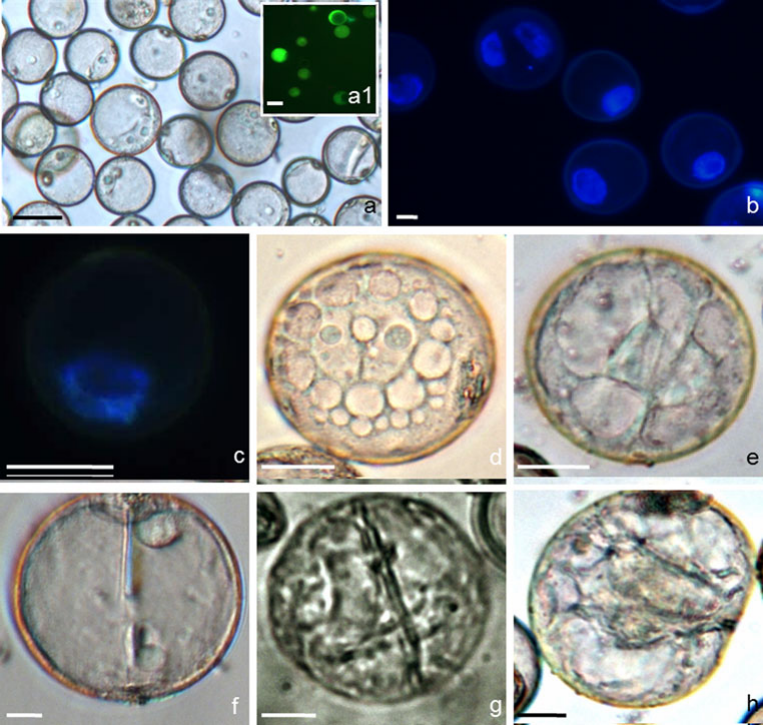
*Miscanthus* × *giganteus* microspore embryogenesis using optimal in vitro culture conditions. **a–c** Uni-nucleate, viable microspores in suspension on the day of isolation, note differences in their size. **d–h** Microspores induced to sporophytic development. Vacuolated microspore with cytoplasmic strands and centrally located nuclei, star-like morphology (**d**). Two-celled structure after symmetrical division with (**e**) or without (**f**) visible cytoplasmic strands. Multicellular structures on day 3 of in vitro culture (**g, h**). Green fluorescence (FITC) by FDA staining demonstrates viable microspores (*a1*). Blue fluorescence (UV) of DAPI demonstrates nuclei (**b, c**).* Bars* 10 μm (**b, d–h**), 20 μm (**a, c**)

Similar to anther culture, all physical treatments (low and high temperatures), chemical treatments (different source of carbohydrates, starvation or *n*-butanol treatment) and co-culture of microspore suspension with immature ovaries, applied in microspore suspension cultures, were unable to promote androgenesis initiation. Although low temperature panicle treatment favored microspore viability and triggered sporophytic development (Fig. 2), the number and quality of androgenic structures obtained was very low.

On isolation day (day 0), microspore suspensions contained mostly viable late uni-nucleate microspores (74.1 %) and immature pollen grains (21.7 %) 20–40 μm in diameter (Fig. 2a–c).

The presence of enlarged microspores was not indicative of further development. The first symmetrical division was preceded by microspore’ vacuolization and the movement of the nucleus to the center of the cell (Fig. 2d). Such uni-nucleate structures with cytoplasmic strands resembled star-like morphology (Fig. 2e). 3.1–6.5 % of the microspores divided mitotically on 190-2 medium supplemented with 500 mg l^−1^ CH, 60 g l^−1^ honey and on 190-2 medium containing low concentration of growth substances (0.375 mg l^−1^ 2,4-D and 0.125 mg l^−1^ kinetin) after 4-days of panicle pretreatment at 10 °C combined with a 3 day low temperature preculture of isolated microspores at 5 °C. After the first symmetrical division, two cells were formed, which were equal in size. The cellularization process was not regular, and in 2 % of the microspores, the newly formed cell wall was not completed after the first division (Fig. 2f). After subsequent mitotic divisions, the number of cells increased, and after about 3 days of culture, multicellular structures were formed within the exine (4.7 %, Fig. 2h). Starting from third week of culture, progressive degeneration of microspores and multicellular structures was observed.

**Fig. 3:**
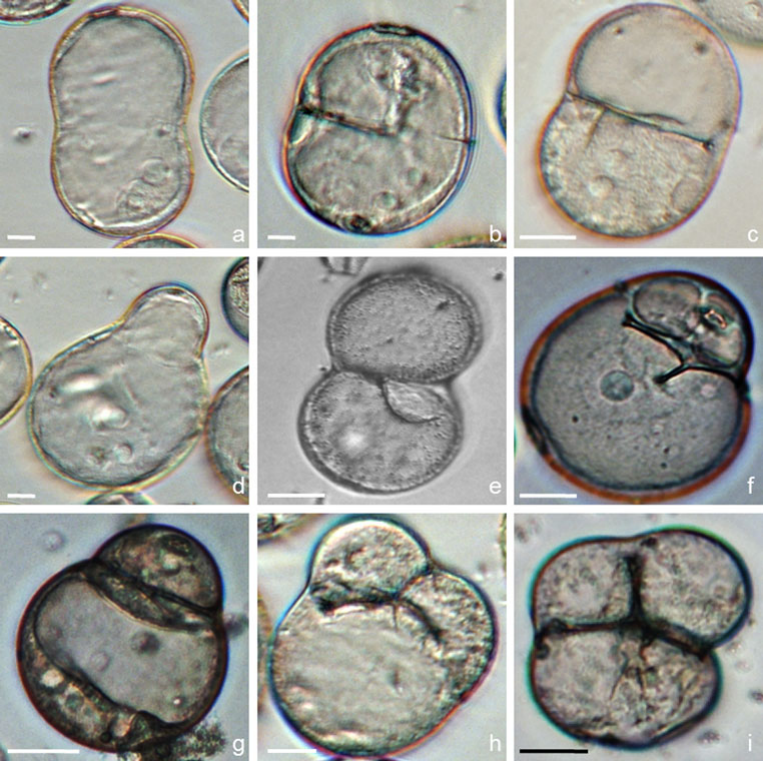
Disturbances in the sporophytic development of *Miscanthus × giganteus* microspores cultured in liquid medium. **a** Bean-shaped uni-nucleated structure. **b–g** Two-celled structures differing in size, shape and organelle content (nuclei, vacuoles) indicate that first mitotic division was not symmetrical (**d, f, g**), and the distribution of the material was not equal to both descendent cells; note that cellularization was not complete (**b, e, f**). **h–i** Three-celled structure after the second mitotic division. Differential interference contrast (DIC),* Bar* 10 μm

As a consequence of prolonged cold stress treatment (14 days at 10 °C of panicle pretreatment followed by 5 days preculture at 4 °C), the sporophytic pathway of isolated microspores was evidently disturbed. The developing microspores differed in size, shape, nuclei number and location, organelle content (e.g., nuclei, vacuoles) and also in the pattern of cellularization (Fig. 3). Bean-shaped uni-nucleated structures with a longer axis about 70 μm in length (Fig. 3a), two-celled abnormal structures with nuclei or cytoplasm and nuclei located in one cell (Fig. 3b–d) and incomplete cellularization (Fig. 3e, f) were observed in suspension cultures. Three-celled androgenic structures were also formed after 3 days of culture. The division was longitudinal to the long axis of developing structures (Fig. 3h, i). Finally, three-celled structures emerged from the exine (Fig. 3i). The transfer to higher temperature (8 °C) caused the degeneration of these structures within 3 days.

Uni-nucleate structures were sporadically formed, which were surrounded by a thick cell wall. Our hypothesis that these structures represented the pollen grains was not confirmed by SEM analysis of pollen grains developed *in planta*.

**Fig. 4:**
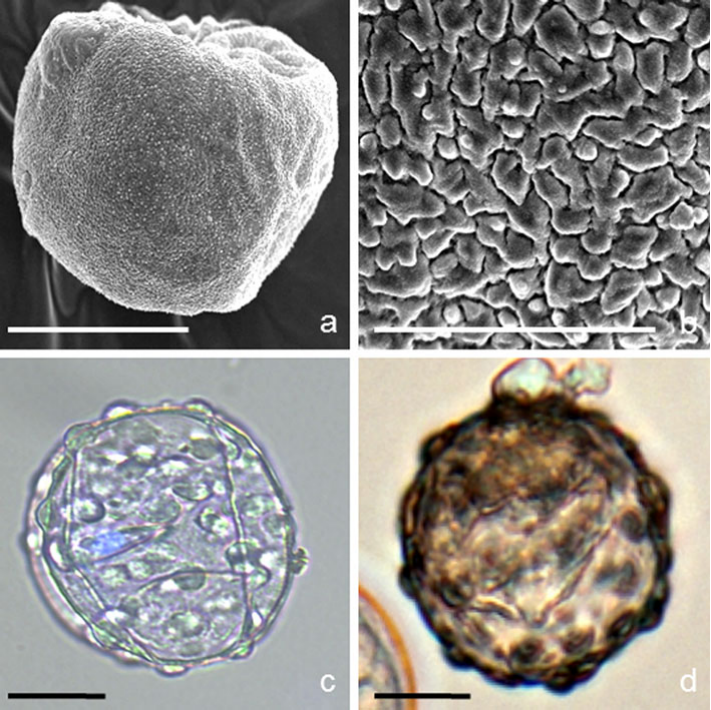
*Miscanthus × giganteus* pollen grains *in planta* and pollen-like structures developed in microspore suspension culture. **a**, **b** Pollen grains *in planta*. Single, monoporate pollen grain **a**. Obscurely verrucate type of the exine sculpture **b**. **c**, **d** Pollen-like structures developing in vitro. Uni-nucleated structures with thick cell wall and incrustation. SEM (**a**, **b**) and in differential interference contrast (DIC, **c, d**) and UV (**c**). Blue fluorescence (UV, DIC) of DAPI demonstrates nucleus. *Bars* 20 μm (**a**), 5 μm (**b**), 10 μm (**c, d**)

SEM analysis revealed single monoporate pollen grains *in planta*. The exine sculpture of pollen grains presented the obscurely verrucate type (Fig. 4a, b), which did not correspond to the images observed in in vitro cultures. (Fig. 4c, d).

**Fig. 5:**
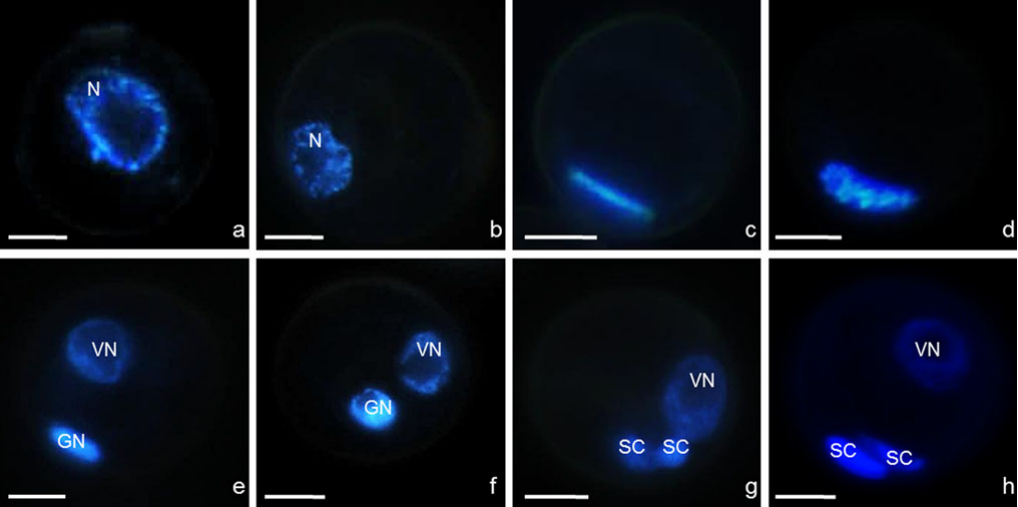
Regular gametogenesis in *Miscanthus × giganteus* of in vitro cultures. Early uni-nucleate microspore with nucleus (*N*) located centrally (**a**). Late uni-nucleate microspore with nucleus (*N*) located close to the sporoderm (**b**). Metaphase (**c**). Anaphase (**d**). Bi-cellular pollen grain with vegetative nucleus (*VN*) and lens-shaped generative nucleus (*GN*) (**e**). Heterochromatin of GN more condensed compare to the heterochromatin of VN. Mid-bi-cellular pollen grain with GN in the vicinity of the VN (**f**). Early stage of three-cellular pollen grain with VN and two sperm cells (*SC*) derived from the GN after the second mitosis. Spherical sperm cell close to the VN (**g**). Late stage of three-cellular pollen grain with VN and two lens-shaped SCs located close to the exine (**h**). Blue fluorescence (UV) of DAPI demonstrates nuclei. *Bar* 10 μm

The majority of in vitro cultured early and late uni-nucleate microspores (Fig. 5a, b) showed the regular gametophytic development and formed pollen-like structures (Fig. 5c, d) The first mitosis (Fig. 5c, d) resulted in the formation of bi-cellular pollen grains with a vegetative (VN) and a generative nucleus (GN). Heterochromatin of the GN was more condensed as compared to the heterochromatin of the VN. The shape of the GN changed from lens-shaped at the early stage to oval at later stages. Initially, the generative cells (GC) were located close to the wall, and the vegetative cell (VC) was much larger and occupied almost the whole space within the sporoderm. Before mitosis II, the GC migrated to a more central position within the VC (Fig. 5e, f). In the vicinity of the VC, the second pollen mitosis of the GC produced two sperm cells (SC, Fig. 5g, h). Finally, tri-cellular pollen-like structures were formed. The ellipsoidal shape of the pollen was similar to that observed in pollen grains filled with starch, which were isolated from freshly cut panicles.

**Fig. 6:**
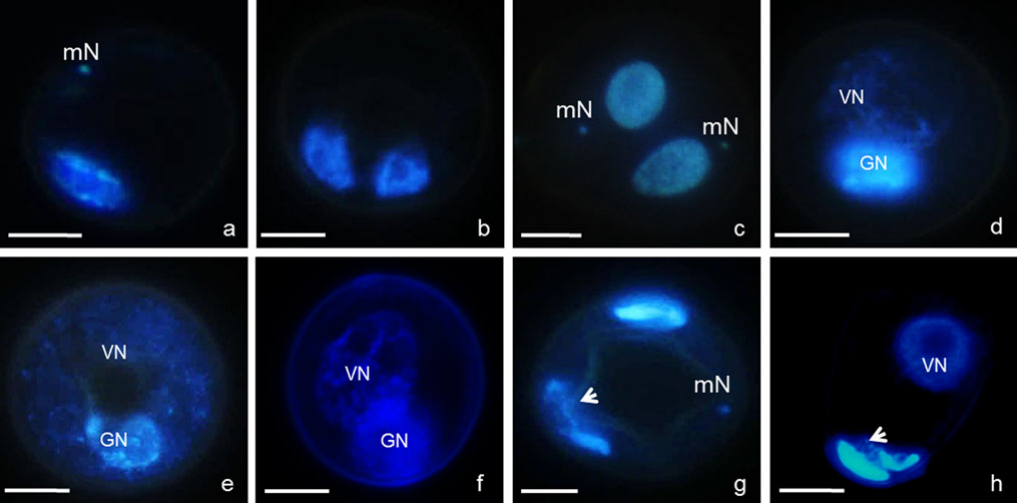
Aberrations in *Miscanthus × giganteus* gametogenesis of in vitro. **a** Anaphase in microspore with micronucleus (mN). **b**, **c** Structure with two equal-sized nuclei without two micronuclei (**b**) and with micronuclei (**c**). **d**, **f** Differences in heterochromatin condensation in bi-cellular structures. Heterochromatin dispersion within VN (**d**, **e**) and within VN and GN (f). **g**, **h** Aberrations during the second pollen mitosis. Anaphase bridge (*arrow*) and micronuclei (**g**). Lagging chromosomes (*arrow*, **h**). Blue fluorescence (UV) of DAPI demonstrates chromatin. *Bar* 10 μm

Abnormal gametophytic pathways under culture conditions led to the production of highly variable pollen-like structures with aberrations in nuclei morphology, heterochromatin condensation and dispersion, micronuclei formation and disturbances in the anaphase (anaphase bridges) of the second mitotic divisions (Fig. 6).

## Discussion

Despite long-standing interdisciplinary research, the mechanism of androgenesis induction remains elusive. A large number of factors, such as genotype, developmental stage of microspores, isolation procedure, composition of media and the stress used for androgenesis induction, determine the usefulness and efficiency of androgenesis (Jähne and Lörz 1995; Touraev et al. 1997; Puolimatka and Pauk 1999; Wang et al. 2000; Shariatpanahi et al. 2006; Wędzony et al. 2009). Different stress pretreatments have been reported to improve the process of androgenesis in various plant species (reviewed in Shariatpanahi et al. 2006; review in Wędzony et al. 2009). In cereals and grasses, high or low temperature shocks have been highly effective. Cold treatment of donor plants, cut tillers or tassels before anther or microspore isolation has been reported to improve androgenic response in barley (Huang and Sunderland 1982; Devaux et al. 1993), wheat (Gustafson et al. 1995; Hu and Kasha 1999), rice (Genovesi and Magill 1979; Chen et al. 1991), maize (Genovesi 1990; Pescitelli et al. 1994) and *Miscanthus sinensis* (Głowacka and Jeżowski 2009). Heat shock has been an effective trigger of androgenesis in wheat (Mejza et al. 1993; Touraev et al. 1996; Hu and Kasha 1999), maize (Genovesi 1990) and rice (Reddy et al. 1985). According to the literature, the most effective treatment to change the microspore developmental pathway is a combination of temperature shock and osmotic/starvation stress achieved by high mannitol concentration of the medium (Ziauddin et al. 1990; Hoekstra et al. 1993; Touraev et al. 1996; Kasha et al. 2001; Zheng et al. 2001; Liu et al. 2002; Cistué et al. 2003; Wędzony et al. 2009; Zur et al. 2008, 2009; Dubas et al. 2010). Stress can be applied during various phases: to donor plants, harvested inflorescences, isolated anthers or microspores. Despite several combinations of these stresses have been extensively investigated in this work, androgenesis initiation was not accomplished neither in anther cultures nor in isolated microspore suspensions of *M.* × *giganteus*. In fact, low temperature (4 °C) and heat shock (30–32 °C) had detrimental effects on *M.* × *giganteus* microspore viability. An initiation of sporophytic development in microspore suspensions using 7 days at 4 °C or panicle incubation at 10 °C suggests that the low temperature stress may promote androgenesis, but the range of temperatures should be chosen so that viability is not reduced. Another important factor, the medium composition, has to be precisely balanced with respect to many parameters, e.g. nutrients, source of carbohydrates, hormones, pH, osmotic potential, gelling agents, etc. (review in Wędzony et al. 2009). All synthetic media tested on *M.* × *giganteus,* C17, KFWC, 190-2, are commonly used in androgenic cultures of monocotyledonous plants and known to support cell growth and to stimulate cell divisions. Among them, C17 and 190-2 seem to create the best environment for anther and isolated microspore culture, respectively. In androgenic cultures, maltose is the most commonly applied source of organic carbon, while also acting as an osmoticum (Hunter 1987; Stephen et al. 1993; Indrianto et al. 1999; Mejza et al. 1993; Wędzony et al. 2009). However, the positive effect of honey in the induction and regeneration of immature inflorescence-derived callus *M.* × *giganteus* has been demonstrated recently by Płażek and Dubert (2010). This effect seems to be confirmed in *M.* × *giganteus* isolated microspore cultures. Honey is essentially a highly concentrated water solution of two sugars, fructose and glucose, with small amount of at least 22 other more complex sugars (White et al. 1980). It contains also various hormones, enzymes, vitamins, mineral salts, free amino acids, and antioxidative phenolics (flavonoids, phenolic acids) and ascorbate. Those last antioxidative compounds (phenolics and ascorbate) are known as preventers of tissue browning (Hołderna-Kędzia and Kędzia 2006) and are presumably responsible for observed effects.

No significant effect on the androgenesis initiation was exerted by the combinations of growth regulators. In contrast, AGPs applied in the frame of an antidesiccation procedure seem to have a positive effect. Although the precise role of AGPs is unclear (Seguí-Simarro et al. 2011), it seems that these compounds play an important function during embryogenesis in suspension cultures, e.g. in *Daucus carrota* (L.) (Marcel et al. 1997; Oxley and Bacic 1999), *Beta vulgaris* (L) (Capataz-Tafur et al. 2011), cotton (Poon et al. 2012) and *Cucurbita pepo pepo* (Amar et al. 2010) as well as in isolated microspore cultures of maize (Borderies et al. 2004), wheat (Letarte et al. 2006), *Brassica napus* (Tang et al. 2006) and white cabbage (Yuan et al. 2012).

Cell enlargement, star-like and symmetrical division are supposed to be some of the early morphological markers for the switch of microspores toward embryogenesis (Touraev et al. 1997; Simmonds and Keller 1999; Shariatpanahi et al. 2006; Dubas et al. 2010, 2011). However, their presence is not tantamount to success as additional restrictions could affect the final result. The problems of ELS’ abortion or the lack of ELS’ regeneration ability are well known (Zheng 2003).

The failure of androgenic embryo development under in vitro conditions in *M. *×* gigantheu*s could result from the cytologically unbalanced nature of microspores, but also from a suboptimal pretreatment of the material and culture media combinations. In this triploid taxon, meiosis is disturbed, resulting in the formation of microspores with different chromosome numbers, differing in size and viability (Słomka et al. 2012). Cytological observations of microspore suspension cultures provided insight into their sporophytic development. In this study, the majority of microspores did not proceed through the regular sporophytic pathway, and they started on the gametophytic pathway or degenerated. It is a well-known phenomenon that the transition from the gametophytic to the sporophytic pathway under in vitro conditions is accompanied by alterations of the cell cycle including the symmetry of cell division, reorganization of the cytoskeleton, vacuolization or cellularization (e.g., Rodríguez-Garcia et al. 2000; Smýkal 2000 and references herein). Similar to *M.* × *giganteus*, heterogeneity of microspore cultures was observed in suspension cultures of cytologically balanced microspores formed after regular male meiosis, e.g. in *Zea mays* (Góralski et al. 2005 and references herein) or in anther culture of *Triticum* (Konieczny et al. 2003). However, androgenic embryos were not produced either in microspore or in anther culture of *M.* × *giganteus*.

## Conclusions

Our results indicate that the induction of androgenesis in vitro in *M.* × *giganteus* is possible. However, the very low efficiency of the process and the absence of regeneration of the produced androgenic structures prevent the practical use of this technique. The cytological analysis suggests that the cause of androgenic recalcitrancy is the hybrid origin of *M.* × *giganteus* deriving from interploidy crosses, resulting in irregular male meiosis.
